# An Artificial MnWO_4_ Cathode Electrolyte Interphase Enabling Enhanced Electrochemical Performance of δ-MnO_2_ Cathode for Aqueous Zinc Ion Battery

**DOI:** 10.3390/ma16083228

**Published:** 2023-04-19

**Authors:** Hao Tian, Huanlin Zhang, You Zuo, Lei Ling, Tengfei Meng, Hang Zhang, Xiaohong Sun, Shu Cai

**Affiliations:** Key Laboratory of Advanced Ceramics and Machining Technology of Ministry of Education, School of Materials Science and Engineering, Tianjin University, Tianjin 300072, China

**Keywords:** cathode electrolyte interphase, Mn dissolution, δ-MnO_2_ cathode, electrochemical performance, aqueous zinc ion battery

## Abstract

The dissolution of active material in aqueous batteries can lead to a rapid deterioration in capacity, and the presence of free water can also accelerate the dissolution and trigger some side reactions that affect the service life of aqueous batteries. In this study, a MnWO_4_ cathode electrolyte interphase (CEI) layer is constructed on a δ-MnO_2_ cathode by cyclic voltammetry, which is effective in inhibiting the dissolution of Mn and improving the reaction kinetics. As a result, the CEI layer enables the δ-MnO_2_ cathode to produce a better cycling performance, with the capacity maintained at 98.2% (vs. activated capacity at 500 cycles) after 2000 cycles at 10 A g^−1^. In comparison, the capacity retention rate is merely 33.4% for pristine samples in the same state, indicating that this MnWO_4_ CEI layer constructed by using a simple and general electrochemical method can promote the development of MnO_2_ cathodes for aqueous zinc ion batteries.

## 1. Introduction

At present, aqueous zinc ion batteries (AZIBs) have been widely studied for energy storage due to various advantages such as low cost, environmental benignity, and high safety performance [1,2,3,4,5,6,7,8,9,10]. So far, there have been various materials reported to be suitable as cathodes for AZIBs, including manganese-based materials [11,12,13,14,15], vanadium-based materials [16,17,18,19,20], and Prussian blue analogs [21,22,23,24]. Among them, manganese oxide has attracted widespread attention because of its abundance, low toxicity, high energy density, and structural diversity [25]. However, Mn dissolution issues still limit the cycling stability of manganese-based materials for AZIBs [26].

In the past decades, there has been some progress made in the research on how to improve the performance of electrode materials [27] and electrolytes [28] in resisting the dissolution of cathodes. Apart from the existing strategies, researchers have also discovered that the construction of an electrode-electrolyte interphase (EEI), including anode electrolyte interphase and cathode electrolyte interphase, can also help improve the energy density, cycling performance, and power density of batteries [29]. Therefore, building the cathode electrolyte interphase (CEI) surface protection layer is considered a feasible solution to the dissolution of cathodes. However, the research on CEI is still limited now due to the complexity of the cathode energy storage mechanism for AZIBs and the difficulty in characterizing various interfacial reactions [30].

Typically, the methods of CEI construction are divided into two categories: in situ formation and artificial synthesis. In recent years, some studies have reportedly been conducted on the in situ formation of CEI. Liang et al. proposed to perform electrochemical synthesis of the CaSO_4_·2H_2_O layer in situ on a Ca_2_MnO_4_ cathode, which significantly improved the stability and service life of the battery [31]. Cao et al. also built an in situ CEI layer of BaSO_4_ on the Ba_0.26_V_2_O_5_·0.92H_2_O cathode of AZIBs, which reduced the dissolution of cathodes, thus leading to an excellent cycling performance [32]. Compared with the synthesis of CEI in situ, the artificial construction of CEI is much easier to manipulate. Xiong et al. synthesized a reduced graphene oxide (rGO) layer coated with α-MnO_2_ powder, which improved both rate performance and cycling stability [33]. For different coating materials, powder coating may cause hindrances to ion transport to some extent. Unlike the powder coating as described above, the artificial CEI is more similar to cathode coating, where a layer is formed between the cathode and the electrolyte. Guo et al. reported a H_f_O_2_ layer formed on a Zn_3_V_2_O_7_(OH)_2_·2H_2_O electrode as an artificial solid electrolyte interphase. The H_f_O_2_ layer was built by means of atomic layer deposition and is capable of isolating the electrode from the electrolyte, thereby reducing the dissolution of the cathode in the electrolyte and inhibiting the formation of insulated by-products. As a result, the capacity retention rate was considerably improved from 45% to 90% after 100 cycles at 0.1 A g^−1^ [34]. Based on the construction of the CEI layer and its effect on cycling performance, there have been many studies carried out. For example, Paraffin [35], (Zn(OH)_2_)_3_(ZnSO_4_)(H_2_O)_5_ [36], and SrCO_3_ [37] have been reported as a kind of CEI layer. All these results demonstrate that the construction of CEI is effective in significantly enhancing the electrochemical performance of AZIBs.

For the manganese-based material used in AZIBs, the CEI layer can help prevent the cathode from direct exposure to the electrolyte, which suppresses Mn dissolution, thus maintaining high cycling performance and capacity. However, the strategy of CEI layer construction on manganese-based materials is not universal, and the economic benefits are unsatisfactory. In this study, a CEI layer of MnWO_4_ is constructed on a δ-MnO_2_ cathode through a facile electrochemical method (cyclic voltammetry), which not only inhibits the Mn dissolution but also improves the reaction kinetics. The key to this economical and efficient strategy lies in the dissolution of the Mn-based cathode in solutions that do not co-exist with Mn^2+^ ions. The prepared MnWO_4_-coated δ-MnO_2_ (denoted as W-MnO_2_) shows an outstanding cycling performance (98.2% capacity retention vs. activated capacity at 500 cycles, after 2000 cycles at 10 A g^−1^), indicating the effectiveness of the CEI construction strategy. In addition, the low-cost strategy of the CEI layer can be applied to other manganese-based AZIBs.

## 2. Experimental Section

### 2.1. Materials Preparation

The synthesis of the cubic MnCO_3_ precursor was performed in the way as reported by others [38]. Firstly, 25 mL of 0.8 M NH_4_HCO_3_ aqueous solution, 25 mL of n-butanol, and 500 mL of cyclohexane were thoroughly mixed. Then, 20 g of cetyltrimethylammonium bromide (CTAB) was added into the mixed solution and stirred until it became clear. Next, 25 mL of 0.4 M MnSO_4_ aqueous solution was added dropwise into the solution, which led to a white precipitate. Afterwards, the precipitate was collected through centrifugation, washed clean with alcohol and distilled water, respectively, and dried under vacuum at 100 °C to obtain white MnCO_3_. To further oxidize MnCO_3_ into MnO_2_ [39,40], 1 g of the synthesized MnCO_3_ precursor was added into 0.032 M of KMnO_4_ aqueous solution. Then, the mixed solution was subjected to ultrasonic treatment for 30 min and stirred for 1 day. The δ-MnO_2_ precursor was collected by centrifugation, washed (three times) with alcohol and distilled water, and finally dried at 75 °C.

The δ-MnO_2_ cathode was produced by using N-methylpirpiridone (NMP) as a solvent to disperse the precursor powder (δ-MnO_2_, 70 wt%), conductive additive (Super P, 20 wt%), and binder (PVDF, 10 wt%), and was coated on carbon fiber paper. The loading density of the cathode was set to about 1.5 mg cm^−2^.

The MnWO_4_-coated δ-MnO_2_ (W-MnO_2_) was constructed in a conventional three-electrode configuration by cyclic voltammetry (CV) at a scan rate of 50 mV/s (negative scan from −0.6 to 0.6 V for 100 segments). The electrolyte was 0.1 mol/L Na_2_WO_4_ solution, and the pH value was adjusted to 7 by using H_2_SO_4_. The pristine δ-MnO_2_ cathode was treated as the working electrode of the three-electrode system, while Ag/AgCl and graphite were taken as the reference and counter electrodes, respectively. Finally, W-MnO_2_ was obtained by washing it thoroughly with distilled water (three times) and drying it at 75 °C. The loading density of the W-MnO_2_ cathode was approximately 0.5–1% higher than the pristine cathode.

### 2.2. Materials Characterization

X-ray diffraction (XRD, D8 Advance, Bruker, Cu Kα) data were collected at a scan range of 5–70° (2θ) and a step size of 0.02°. Both SEM (Sigma 300, Zeiss, operating voltage 5 kV) and HR-TEM (JEM-2100F, JEOL) were employed to examine the morphology and microstructure of the samples. X-ray photoelectron spectroscopy (XPS, PHI-1600, PerkinElmer) was performed to record the valence states of the samples loaded with Cu. The C 1s peak with a binding energy of 284.8 eV was used to calibrate all XPS spectra. Nitrogen adsorption measurements for Brunauer–Emmett–Teller (BET) analysis were tested at 77 K using an ASAP 2460.

### 2.3. Electrochemical Measurements

The 2032-type coin cells were assembled with prepared W-MnO_2_ as the cathode, a Zn foil as the counter electrode, and an aqueous ZnSO_4_ (3 M) solution with a MnSO_4_ additive (0.2 M) as the electrolyte. The CV and EIS (100 kHz to 10 mHz) were measured on a CHI 660E electrochemical workstation. To conduct the CV tests at different scan rates, the peak current (i) and scan rate (ν) were determined through Equation (1) [41]:

(1) $$i={a\nu }^{b},$$

where a and b represent variable parameters, and the b-value is obtained through the slope of log(i) vs. log(ν). Furthermore, the current contribution is divided into capacitive and diffusion contributions according to Equation (2) [42]:

(2) $$i=k_{1}V+k_{2}V^{\frac{1}{2}},$$

where k_1_ and k_2_ refer to the coefficients of proportionality for capacitive and diffusion contributions, respectively. The GCD curves, cycling performance, and GITT measurements were achieved by using the LAND CT2001A battery test system at room temperature. Moreover, the pause and rest time of GITT at 0.2 A g^−1^ lasted 10 min and 180 min, respectively. The diffusion coefficient can be determined through the following equation [43]:

(3) $$D=\frac{4l^{2}}{\pi \tau },$$

where D represents the diffusion coefficient, l indicates the diffusion length (cm) of active materials, and τ refers to the duration of the current pause (s). ΔE_s_ and ΔE_t_ represent the voltage difference by the current pulse and the voltage difference during the constant current pulse, respectively.

## 3. Results and Discussion

**Figure 1:**
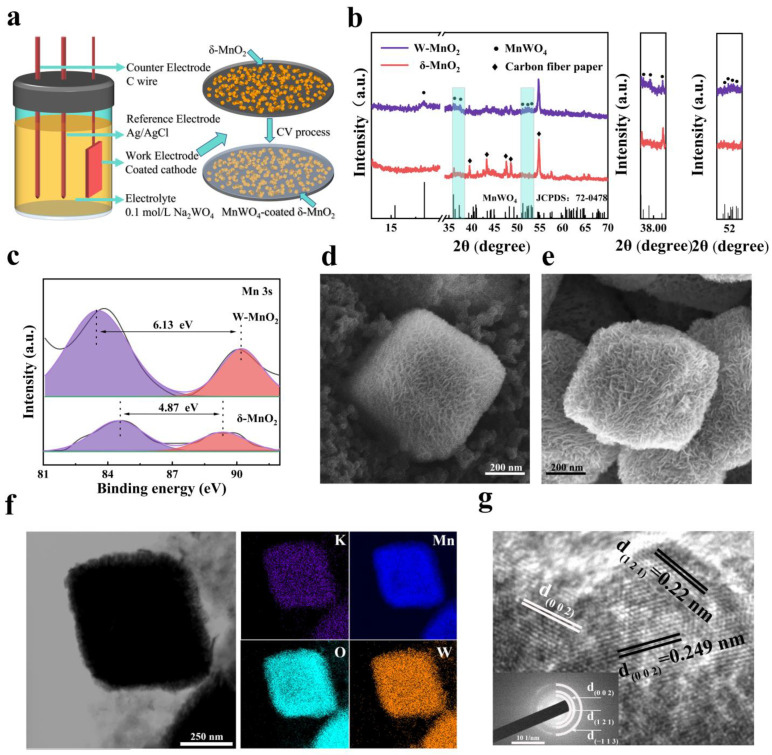
(**a**) Schematic illustration of the preparation process of MnWO_4_ CEI layer. (**b**) XRD patterns of δ-MnO_2_ and W-MnO_2_. (**c**) High-resolution XPS spectra of Mn 3s region in δ-MnO_2_ and W-MnO_2_. (**d**,**e**) The morphology of (**d**) δ-MnO_2_ and (**e**) W-MnO_2_. (**f**) TEM image and the elemental mappings of W-MnO_2_. (**g**) HRTEM image of MnWO_4_ on the surface of W-MnO_2_ and the corresponding SAED pattern (inset).

The MnWO_4_-coated δ-MnO_2_ (W-MnO_2_) was obtained by means of the electrochemical treatment (cyclic voltammetry) conducted in a three-electrode system, as shown in Figure 1a. The working electrode was the δ-MnO_2_ cathode. Figures S1 and S2 show the XRD and BET results of δ-MnO_2_ powder, respectively. According to the N_2_ adsorption isotherm, the specific area of δ-MnO_2_ is 20 m^2^ g^−1^. To confirm the chemical composition of the CEI layer on the δ-MnO_2_ cathode, XRD was performed for the W-MnO_2_ cathode, as shown in Figure 1b. In addition to the weak characteristic peaks of δ-MnO_2_, a peak appears at 18° corresponding to MnWO_4_ (JCPDS No. 72-0478) after the CV process. Moreover, there are some other characteristic peaks of MnWO_4_ observed at around 37° and 52°, indicating the presence of the MnWO_4_ after the CV process. It is suspected that the absence of the δ-MnO_2_ characteristic peaks may result from the limited crystallinity of δ-MnO_2_ and the strong diffraction peak of the carbon fiber paper. XPS was performed to determine the Mn valence during the CV process. As shown in Figure 1c, the splitting magnitude of two splitting components for the Mn 3s peak increases to 6.13 eV from 4.87 eV after the electrochemical treatment. In general, the Mn 3s peak consists of two multiple splitting components [11,44], with the oxidation state of Mn determined by the splitting magnitude ΔE, which is 6.0 eV and 4.7 eV for Mn^2+^ and MnO_2_ (Mn^4+^), respectively [45]. It can be found that the valence state of Mn shifted from +4 in δ-MnO_2_ to +2 in W-MnO_2_, indicating the formation of the MnWO_4_ on the δ-MnO_2_ cathode. To determine the effect of MnWO_4_ formation on the morphology of δ-MnO_2_ and the area of MnWO_4_ distribution, SEM and TEM tests were performed. As shown in Figure 1d, the size of δ-MnO_2_ cubes is approximately 500 nm, and the morphology of the δ-MnO_2_ cubes is barely changed during the formation of MnWO_4_ (Figure 1e). The EDS element mapping of W-MnO_2_ (Figure 1f) shows a uniform distribution of element W on the surface of the MnO_2_ cube, indicating that the MnWO_4_ is formed uniformly on the surface of the δ-MnO_2_ cathode. Moreover, the HRTEM (Figure 1g) images of the W-MnO_2_ surface show that lattice fringes are 0.22 nm and 0.249 nm, which correspond to the (121) and (002) crystal planes of MnWO_4_, respectively. Meanwhile, the (121), (002), and (−113) crystal planes of MnWO_4_ are also observable in the results of selected area electron diffraction (SAED). Judging from the image of TEM (Figure 1f), it can be concluded that MnWO_4_ was formed on the surface of δ-MnO_2_ cathodes as a CEI layer. Thus, it can be inferred that during the CV process, the δ-MnO_2_ surface is partially dissolved and rapidly reacts with WO_4_^2-^ to form MnWO_4_ during the CV process. Finally, the MnWO_4_ CEI layer is successfully constructed on the δ-MnO_2_ cathode.

**Figure 2:**
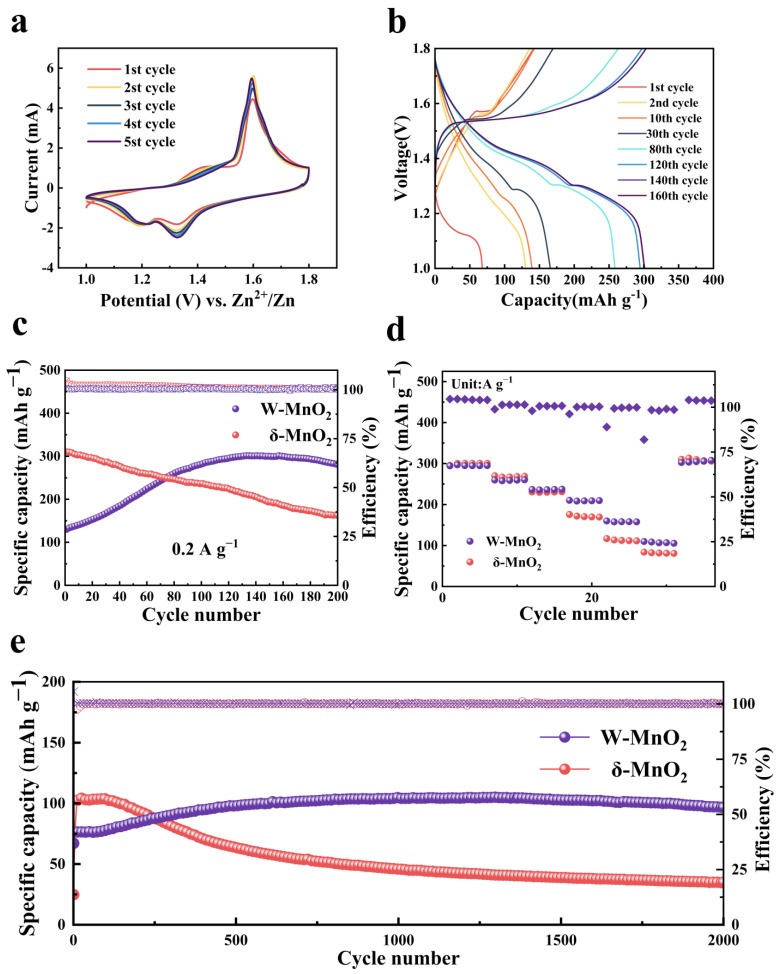
Electrochemical performance of δ-MnO_2_ and W-MnO_2_ cathodes. (**a**) CV curves of W-MnO_2_ cathode at 1 mV s^−1^. (**b**,**c**) Galvanostatic charge and discharge curves of the W-MnO_2_ cathode and corresponding cycling performance at 0.2 A g^−1^. (**d**) Rate capacity of δ-MnO_2_ and W-MnO_2_ cathodes with current density from 0.2 to 10 A g^−1^. (**e**) Cycling performance at 10 A g^−1^.

To examine the effect of the MnWO_4_ CEI layer on the electrochemical performance of the δ-MnO_2_ cathode, a number of coin cells were assembled with 3 M ZnSO_4_ + 0.2 M MnSO_4_ as the electrolyte and zinc foil as the anode. Figure 2a presents the CV curves drawn for the W-MnO_2_ cathode in the initial five CV cycles. The peak of the CV curves almost overlap, and their intensity increases at a slow pace after the second cycle, indicating that the W-MnO_2_ cathode maintains excellent performance in electrochemical activity and reversibility after the construction of the CEI layer. For the W-MnO_2_ cathode, the two cathodic peaks at 1.2 V and 1.4 V correspond to different stages of charge carrier insertion [15,46]. By drawing a comparison with the CV curves of the δ-MnO_2_ cathode (Figure S3), the increased intensity of cathodic peak shown by W-MnO_2_ near 1.4 V is suspected to result from the improvement of reaction kinetics by the MnWO_4_ CEI layer. The galvanostatic charge and discharge (GCD) curve of the W-MnO_2_ cathode at 0.2 A g^−1^ (Figure 2b) shows a slow-paced improvement of capacity during cycling, suggesting the activation of the W-MnO_2_ cathode. Afterwards, the W-MnO_2_ cathode exhibits two-stage charge carrier intercalation, which is coherent with the CV results. In comparison with the GCD curves of the δ-MnO_2_ cathode (Figure S4) and W-MnO_2_ cathode, there is almost no difference found between them, indicating that the CEI layer did not change the characteristics of the two-stage charge carrier intercalation. As confirmed by the cycling test conducted at 0.2 A g^−1^ (Figure 2c), the W-MnO_2_ cathode is slowly activated by the MnWO_4_ CEI layer. In the first 100 cycles, the capacity of the W-MnO_2_ cathode improves slowly and stabilizes gradually at around 301.2 mAh g^−1^, which is close to the initial capacity of the δ-MnO_2_ cathode. However, the capacity of the δ-MnO_2_ cathode declines continuously, which indicates that the MnWO_4_ CEI layer improves the cycling stability significantly. As shown in Figure 2d, the rate capability of the W-MnO_2_ cathode was evaluated after the activation process. To be specific, the W-MnO_2_ cathode achieves a specific discharge capacity of 295.2, 260.5, 237.4, 210.3, 158.1, and 105.5 mAh g^−1^ at the current density of 0.2, 0.5, 1, 2, 5, and 10 A g^−1^, respectively. The corresponding GCD curves of W-MnO_2_ and δ-MnO_2_ cathodes at various current densities are presented in Figures S5 and S6, respectively. Compared with the corresponding values of the δ-MnO_2_ cathode that vary from 0.2 to 10 A g^−1^, the capacity rate of W-MnO_2_ and δ-MnO_2_ cathodes reaches 35.7% and 26.9%, respectively. It implies that the MnWO_4_ CEI layer is conducive to improving rate performance. Notably, the W-MnO_2_ cathode achieves an outstanding performance in cycling stability at 10 A g^−1^, as shown in Figure 2e, from which it can be seen that the capacity of the W-MnO_2_ cathode slowly increases to 98.6 mAh g^−1^ during activation (initial 500 cycles). After 2000 cycles, the W-MnO_2_ cathode maintains a capacity retention rate of 98.2% (vs. the activated capacity at 500 cycles). However, the cycling capacity of the δ-MnO_2_ cathode without the MnWO_4_ CEI layer decreases rapidly after the initial 100 cycles. Subsequently, the capacity is gradually reduced to 35 mAh g^−1^ after 2000 cycles. At this point, the capacity retention rate is merely 33.4%. This result confirms that the electrochemical cycling performance can be improved by the MnWO_4_ CEI layer on the δ-MnO_2_ cathode. Without any significant change in the structure and morphology of the cathode material, a thin layer constructed on the cathode surface is sufficient to improve the electrochemical performance significantly. The construction of the CEI layer is more universal than the adjustment for electrodes [12] and electrolytes [13]. In comparison with other reported CEI or SEI layers (Table S1, Supporting Information) [31,32,33,34,35,36,37], the MnWO_4_ CEI layer, as constructed in this paper, leads to a significant improvement of high current cycle performance. 

**Figure 3:**
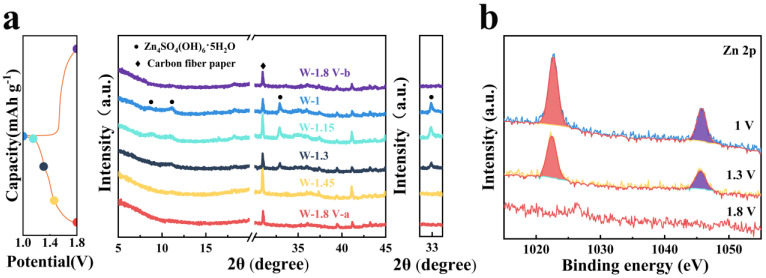
(**a**) Ex situ XRD analysis of W-MnO_2_ cathode at various voltages. (**b**) XPS spectra of Zn 2p at different discharge stages.

To explore the effect of the CEI layer on the mechanism of energy storage, the structure evolution of the W-MnO_2_ cathode was analyzed by means of ex situ XRD, XPS, and TEM. Taking into account the BET result of δ-MnO_2_ and the morphology change of the W-MnO_2_ cathode, the storage mechanism was analyzed through bulk diffusion rather than surface adsorption. The ex situ XRD of the W-MnO_2_ cathode (Figure 3a) reveals the incremental increase of characteristic peaks (around 10° and 33°) corresponding to Zn_4_SO_4_(OH)_6_·xH_2_O upon the entire discharge process, which evidences the occurrence of H^+^ insertion. This is consistent with the findings of previous research [47,48,49,50]. In addition, the formation of Zn_4_SO_4_(OH)_6_·xH_2_O nanosheet at the discharge stage is revealed by ex situ TEM (Figure S7). The results of TEM mapping show the presence of S, Zn, and O elements. Moreover, it can be seen from the SAED pattern (Figure S7) that the nanosheet is Zn_4_SO_4_(OH)_6_·xH_2_O. In contrast, H^+^ is gradually released from the W-MnO_2_ cathode during the subsequent charge to 1.8 V, which is accompanied by the disappearance of Zn_4_SO_4_(OH)_6_·xH_2_O, as shown in Figure 3c. That is to say, reversible (de)insertion occurs to H^+^ throughout the storage process. The reversible storage of Zn^2+^ in the W-MnO_2_ host is confirmed by the ex situ XPS performed on the acid-washed cathodes (Figure 3b). When the cathode is discharged to 1.3 V, there are two strong peaks emerging at 1045.8 and 1022.7 eV, which can be considered evidence of Zn^2+^ intercalation [51,52]. The peak strength of the Zn 2p further increases when the cathode is fully discharged (1 V), indicating the occurrence of Zn^2+^ intercalation throughout the discharge process. In addition, the stability of the MnWO_4_ CEI film during the cycle process is indicated by TEM, HRTEM, and corresponding SAED (Figure S8) in full charge and discharge states. Therefore, H^+^/Zn^2+^ co-insertion is confirmed as the storage mechanism of the W-MnO_2_ cathode during the discharge process.

**Figure 4:**
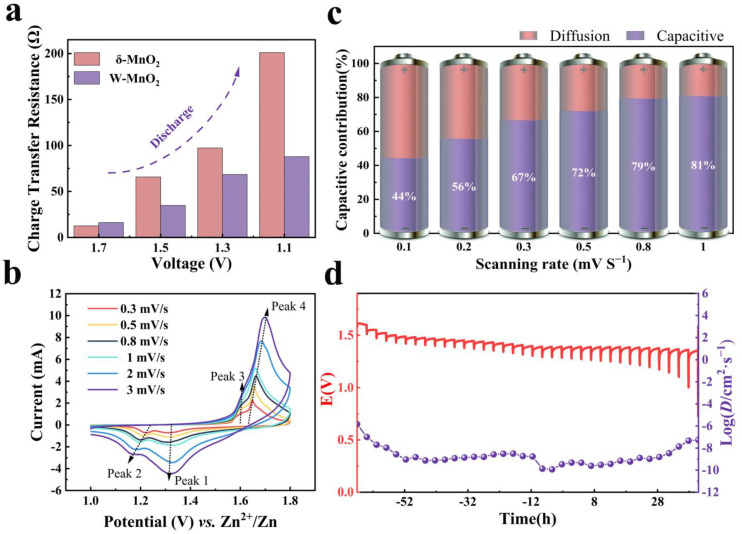
(**a**) The charge transfer resistance of W-MnO_2_ and δ-MnO_2_ cathodes at the discharge stage. (**b**) CV tests at various scan rates from 0.3 to 3 mV s^−1^. (**c**) The proportion of capacitive contributions at different scan rates for W-MnO_2_. (**d**) GITT curves of the W-MnO_2_ cathode after activation during the discharge process.

When the mechanism of energy storage is investigated, the reversible formation of the by-product (Zn_4_SO_4_(OH)_6_·xH_2_O) on the cathode is worth noting. During the discharge process, the formation of Zn_4_SO_4_(OH)_6_·xH_2_O nanosheets could inhibit the electrochemical reaction in the cathode to some extent, as reported in other studies [34]. To demonstrate the impact of the by-product (Zn_4_SO_4_(OH)_6_·xH_2_O) on the charge transfer resistance, the EIS test was performed during the discharge process. The EIS data (Figure S9) of the W-MnO_2_ cathode were fitted with the equivalent circuit template, as indicated by two semicircles in the medium and high-frequency regions. The semicircle at a high frequency is considered as the constructed MnWO_4_ CEI layer and the Zn_4_SO_4_(OH)_6_·xH_2_O formed during the discharge process, while that at a medium frequency is attributed to the charge transfer resistance (R_ct_). Figure 4a shows the variation and comparison of the R_ct_ during different stages of discharge for both W-MnO_2_ and δ-MnO_2_ cathodes. Apparently, the R_ct_ of δ-MnO_2_ cathode increases rapidly (from 12.62 to 201 Ω), which suggests that the existence of Zn_4_SO_4_(OH)_6_·xH_2_O nanosheet plays a part in insulating the active material, which impedes electron transport and increases internal resistance. For the W-MnO_2_ cathode, the incremental of R_ct_ is more significant compared to the δ-MnO_2_ cathode, indicating that the impact of Zn_4_SO_4_(OH)_6_·xH_2_O is mitigated by the presence of the MnWO_4_ CEI layer. Moreover, the R_ct_ of the W-MnO_2_ cathode is higher than that of the δ-MnO_2_ cathode in the initial state, which is due to the relatively low conductivity of the MnWO_4_ CEI layer. 

To reveal the effect of the MnWO_4_ CEI layer and Zn_4_SO_4_(OH)_6_·xH_2_O intermediate on the reaction kinetics of the W-MnO_2_ cathode, the kinetics behaviors were analyzed by carrying out CV (cyclic voltammetric curve) tests at varying scan rates (0.3 to 3.0 mV s^−1^), as shown in Figure 4b. For the W-MnO_2_ cathode, the b-value (Figure S11a) of the three different peaks is calculated to be 0.80, 0.58, and 0.62 for peaks 1, 2, and 4, respectively. As for δ-MnO_2_ cathode (Figures S10 and S11b), the b-value of peaks 1, 2, and 4 is 0.6, 0.41, and 0.53, respectively. The rise in the b-value of the W-MnO_2_ cathode indicates that the improvement of reaction kinetics contributes to an excellent rate performance [53]. Furthermore, the capacitive-controlled contribution for the W-MnO_2_ cathode is calculated to be 44.1%, 55.5%, 66.5%, 71.9%, 79.2%, and 80.7% at a scan rate of 0.1, 0.2, 0.3, 0.5, 0.8, and 1 mV s^−1^, respectively (Figure 4c). The proportion of capacitive contribution to the whole capacity for the W-MnO_2_ cathode at 1 mV s^−1^ is 80.7%, suggesting that the pseudocapacitive behavior dominates the storage mechanism. Compared with the δ-MnO_2_ cathode, the capacitive contributions of the δ-MnO_2_ cathode (Figure S12) is less significant at different scan rates, which reaffirms the improvement of reaction kinetics by the construction of the MnWO_4_ CEI layer. Finally, to gain an insight into the diffusion dynamics, the galvanostatic intermittent titration technique (GITT) was applied to calculate the diffusion coefficient (D) at different stages of discharge (Figure 4d and Figure S13). It can be found that the W-MnO_2_ and δ-MnO_2_ cathodes experience two stages of discharge according to the D value. In the first one, D is between 10^−8^ and 10^−9^. In the second one, D decreases to the range of 10^−9^–10^−10^. It is noteworthy that the MnWO_4_ CEI layer causes the diffusion coefficient of the W-MnO_2_ cathode to be relatively more stable, which is always above 10^−10^. To sum up, the MnWO_4_ CEI layer of δ-MnO_2_ can mitigate the impact of Zn_4_SO_4_(OH)_6_·xH_2_O on the cathode and ensure sufficient reaction kinetics, which explains the better electrochemical performance.

## 4. Conclusions

In the present study, a MnWO_4_ CEI layer was constructed on the δ-MnO_2_ surface by following a facile cyclic voltammetry method, which significantly reduced the impact of by-product (Zn_4_SO_4_(OH)_6_·xH_2_O) on the cathode and improved the reaction kinetics during the process of H^+^/Zn^2+^ co-intercalation, thus enhancing the rate performance (295.2 mA at 0.1 A g^−1^ and 105.5 mA at 10 A g^−1^). More importantly, the dissolution of Mn was inhibited in the AZIBs by the MnWO_4_ CEI layer, thus ensuring its long cycling lifespan. Compared to the activated capacity at 500 cycles, the capacity retention rate at 10 A g^−1^ was maintained at 98.2% after 2000 cycles, which is much higher than the retention rate of 33.4% for the pristine MnO_2_ cathode. This CEI construction strategy could contribute to exploring the stable Mn-based cathode of AZIBS.

## Data Availability

The data presented in this study are available on request from the corresponding author.

## Supplementary Materials

The following supporting information can be downloaded at: https://www.mdpi.com/article/10.3390/ma16083228/s1, Figure S1: XRD pattern of synthetic δ-MnO_2_ pristine powder; Figure S2: N_2_ adsorption isotherms of δ-MnO_2_; Figure S3: CV curves of the δ-MnO_2_ electrode at 1 mV/s; Figure S4: Galvanostatic charge and discharge curves of the δ-MnO_2_ cathode at 0.2 A g^−1^; Figure S5: Galvanostatic charge and discharge curves of W-MnO_2_ cathode at various current densities ranging from 0.2 A g^−1^ to 10 A g^−1^; Figure S6: Galvanostatic charge and discharge curves of the δ-MnO_2_ cathode at various current densities ranging from 0.2 A g^−1^ to 10 A g^−1^; Figure S7: High-angle annular bright-field scanning TEM (HAABF-STEM) image and the corresponding elemental mappings, respectively; Figure S8: (a,b) TEM, HR-TEM and SAED pattern of W-MnO_2_: (a,c) charged (1.8 V). (b,d) discharged (1 V); Figure S9: EIS of (a) the W-MnO_2_ and (b) δ-MnO_2_ cathodes at different stages during the discharge process. The equivalent circuit model for (c) the W-MnO_2_ and d) the δ-MnO_2_ electrode; Figure S10: CV tests for δ-MnO_2_ cathode at various scan rates ranging from 0.3 to 3 mV/s; Figure S11: (a,b) b values of different peaks in CV curves for W-MnO_2_ and δ-MnO_2_ cathodes: (a) W-MnO_2_. (b) δ-MnO_2_; Figure S12: The proportion of capacitive and diffusion contributions at various scan rates for δ-MnO_2_; Figure S13: GITT test of the δ-MnO_2_ cathode; Table S1: Comparison of cycling performance with other recent studies [31,32,33,34,35,36,37].

## References

[1] Macrelli A., Olivieri M., Lamperti A., Russo V., Bozzini B., Menegazzo M., Bussetti G., Casari C.S., Li Bassi A. (2023). Nanostructured Zn_x_Mn_3−x_O_4_ thin films by pulsed laser deposition: A spectroscopic and electrochemical study towards the application in aqueous Zn-ion batteries. Electrochim. Acta.

[2] Zampardi G., La Mantia F. (2020). Prussian blue analogues as aqueous Zn-ion batteries electrodes: Current challenges and future perspectives. Curr. Opin. Electrochem..

[3] Liu Z., Qin L., Cao X., Zhou J., Pan A., Fang G., Wang S., Liang S. (2022). Ion migration and defect effect of electrode materials in multivalent-ion batteries. Prog. Mater. Sci..

[4] Tang B., Fang G., Zhou J., Wang L., Lei Y., Wang C., Lin T., Tang Y., Liang S. (2018). Potassium vanadates with stable structure and fast ion diffusion channel as cathode for rechargeable aqueous zinc-ion batteries. Nano Energy.

[5] Chen D., Lu M., Cai D., Yang H., Han W. (2021). Recent advances in energy storage mechanism of aqueous zinc-ion batteries. J. Energy Chem..

[6] Song M., Tan H., Chao D., Fan H.J. (2018). Recent advances in Zn-ion batteries. Adv. Funct. Mater..

[7] Fegade U., Jethave G., Khan F., Al Ahmed A., Karmouch R., Shariq M., Inamuddin, Ahmer M.F. (2022). Recent development of aqueous zinc-ion battery cathodes and future challenges: Review. Int. J. Energ. Res..

[8] Liu S., Sun Y., Yang J., Zhang Y., Cai Z. (2022). Highly loaded and binder-free molybdenum trioxide cathode material prepared using multi-arc ion plating for aqueous zinc ion batteries. Materials.

[9] Li T., Li H., Yuan J., Xia Y., Liu Y., Sun A. (2022). Recent advance and modification strategies of transition metal dichalcogenides (TMDs) in aqueous zinc ion batteries. Materials.

[10] Karapidakis E., Vernardou D. (2021). Progress on V_2_O_5_ cathodes for multivalent aqueous batteries. Materials.

[11] Wang D., Wang L., Liang G., Li H., Liu Z., Tang Z., Liang J., Zhi C. (2019). A Superior δ-MnO_2_ cathode and a self-healing Zn-δ-MnO_2_ battery. ACS Nano.

[12] Alfaruqi M.H., Islam S., Mathew V., Song J., Kim S., Tung D.P., Jo J., Kim S., Baboo J.P., Xiu Z. (2017). Ambient redox synthesis of vanadium-doped manganese dioxide nanoparticles and their enhanced zinc storage properties. Appl. Surf. Sci..

[13] Chamoun M., Brant W.R., Tai C., Karlsson G., Noréus D. (2018). Rechargeability of aqueous sulfate Zn/MnO_2_ batteries enhanced by accessible Mn^2+^ ions. Energy Storage Mater..

[14] Zuo Y., Meng T., Tian H., Ling L., Zhang H., Zhang H., Sun X., Cai S. (2023). Enhanced H^+^ storage of a MnO_2_ cathode via a MnO_2_ nanolayer interphase transformed from manganese phosphate. ACS Nano.

[15] Sun W., Wang F., Hou S., Yang C., Fan X., Ma Z., Gao T., Han F., Hu R., Zhu M. (2017). Zn/MnO_2_ battery chemistry with H^+^ and Zn^2+^ coinsertion. J. Am. Chem. Soc..

[16] Kundu D., Adams B.D., Duffort V., Vajargah S.H., Nazar L.F. (2016). A high-capacity and long-life aqueous rechargeable zinc battery using a metal oxide intercalation cathode. Nat. Energy.

[17] Chen J., Xiao B., Hu C., Chen H., Huang J., Yan D., Peng S. (2022). Construction strategy of VO_2_ @V_2_C 1D/2D heterostructure and improvement of zinc-ion diffusion ability in VO_2_ (B). ACS Appl. Mater. Inter..

[18] Dong Y., Jia M., Wang Y., Xu J., Liu Y., Jiao L., Zhang N. (2020). Long-life zinc/vanadium pentoxide battery enabled by a concentrated aqueous ZnSO_4_ electrolyte with proton and zinc ion co-intercalation. ACS Appl. Energ. Mater..

[19] Zhang N., Dong Y., Jia M., Bian X., Wang Y., Qiu M., Xu J., Liu Y., Jiao L., Cheng F. (2018). Rechargeable aqueous Zn-V_2_O_5_ battery with high energy density and long cycle life. ACS Energy Lett..

[20] Chen L., Ruan Y., Zhang G., Wei Q., Jiang Y., Xiong T., He P., Yang W., Yan M., An Q. (2019). Ultrastable and high-performance Zn/VO_2_ battery based on a reversible single-phase reaction. Chem. Mater..

[21] Lu K., Song B., Zhang J., Ma H. (2016). A rechargeable Na-Zn hybrid aqueous battery fabricated with nickel hexacyanoferrate and nanostructured zinc. J. Power Sources.

[22] Yang Q., Mo F., Liu Z., Ma L., Li X., Fang D., Chen S., Zhang S., Zhi C. (2019). Activating C-coordinated iron of iron hexacyanoferrate for Zn hybrid-ion batteries with 10,000-cycle lifespan and superior rate capability. Adv. Mater..

[23] Yi H., Qin R., Ding S., Wang Y., Li S., Zhao Q., Pan F. (2021). Structure and properties of Prussian blue analogues in energy storage and conversion applications. Adv. Funct. Mater..

[24] Zhang L., Chen L., Zhou X., Liu Z. (2015). Towards high-voltage aqueous metal-ion batteries beyond 1.5 V: The zinc/zinc hexacyanoferrate system. Adv. Energy Mater..

[25] Zhao Y., Zhu Y., Zhang X. (2020). Challenges and perspectives for manganese-based oxides for advanced aqueous zinc-ion batteries. Infomat.

[26] Ren Q., Yuan Y., Wang S. (2022). Interfacial strategies for suppression of Mn dissolution in rechargeable battery cathode materials. ACS Appl. Mater. Inter..

[27] Fang G., Zhu C., Chen M., Zhou J., Tang B., Cao X., Zheng X., Pan A., Liang S. (2019). Suppressing manganese dissolution in potassium manganate with rich oxygen defects engaged high-energy-density and durable aqueous zinc-ion battery. Adv. Funct. Mater..

[28] Poyraz A.S., Laughlin J., Zec Z. (2019). Improving the cycle life of cryptomelane type manganese dioxides in aqueous rechargeable zinc ion batteries: The effect of electrolyte concentration. Electrochim. Acta.

[29] Zhou M., Chen Y., Fang G., Liang S. (2022). Electrolyte/electrode interfacial electrochemical behaviors and optimization strategies in aqueous zinc-ion batteries. Energy Storage Mater..

[30] Verma V., Kumar S., Manalastas W., Srinivasan M. (2021). Undesired reactions in aqueous rechargeable zinc ion batteries. ACS Energy Lett..

[31] Guo S., Liang S., Zhang B., Fang G., Ma D., Zhou J. (2019). Cathode interfacial layer formation via in situ electrochemically charging in aqueous zinc-ion battery. ACS Nano.

[32] Luo S., Cao X., Su Q., Zhang Y., Liu S., Xie X., Liang S., Pan A. (2021). Layered barium vanadate cathodes for aqueous zinc batteries: Enhancing cycling stability through inhibition of vanadium dissolution. ACS Appl. Energ. Mater..

[33] Wu B., Zhang G., Yan M., Xiong T., He P., He L., Xu X., Mai L. (2018). Graphene scroll-coated α-MnO_2_ nanowires as high-performance cathode materials for aqueous Zn-ion battery. Small.

[34] Guo J., Ming J., Lei Y., Zhang W., Xia C., Cui Y., Alshareef H.N. (2019). Artificial solid electrolyte interphase for suppressing surface reactions and cathode dissolution in aqueous zinc ion batteries. ACS Energy Lett..

[35] Li S., Yu D., Liu L., Yao S., Wang X., Jin X., Zhang D., Du F. (2022). In-situ electrochemical induced artificial solid electrolyte interphase for MnO@C nanocomposite enabling long-lived aqueous zinc-ion batteries. Chem. Eng. J..

[36] Liu Y., Zhi J., Hoang T.K.A., Zhou M., Han M., Wu Y., Shi Q., Xing R., Chen P. (2022). Paraffin Based cathode-electrolyte interface for highly reversible aqueous zinc-ion battery. ACS Appl. Energ. Mater..

[37] Zhang L., Zhang B., Hu J., Liu J., Miao L., Jiang J. (2021). An in situ artificial cathode electrolyte interphase strategy for suppressing cathode dissolution in aqueous zinc ion batteries. Small Methods.

[38] Chen X., Cao Z., Xing L., Liao Y., Qiu Y., Li W. (2017). Improved Li-storage performance with PEDOT-decorated MnO_2_ nanoboxes. Nanoscale.

[39] Guo C., Zhou Q., Liu H., Tian S., Chen B., Zhao J., Li J. (2019). A case study of β- and δ-MnO_2_ with different crystallographic forms on ion-storage in rechargeable aqueous zinc ion battery. Electrochim. Acta.

[40] Wu P., Dai S., Chen G., Zhao S., Xu Z., Fu M., Chen P., Chen Q., Jin X., Qiu Y. (2020). Interfacial effects in hierarchically porous α-MnO_2_/Mn_3_O_4_ heterostructures promote photocatalytic oxidation activity. Appl. Catal. B Environ..

[41] Puttaswamy R., Nagaraj R., Kulkarni P., Beere H.K., Upadhyay S.N., Balakrishna R.G., Sanna Kotrappanavar N., Pakhira S., Ghosh D. (2021). Constructing a high-performance aqueous rechargeable zinc-ion battery cathode with self-assembled mat-like packing of intertwined Ag(I) pre-inserted V_3_O_7_·H_2_O microbelts with reduced graphene oxide core. ACS Sustain. Chem. Eng..

[42] Cook J.B., Kim H.S., Yan Y., Ko J.S., Robbennolt S., Dunn B., Tolbert S.H. (2016). Mesoporous MoS_2_ as a transition metal dichalcogenide exhibiting pseudocapacitive Li and Na-ion charge storage. Adv. Energy Mater..

[43] Deiss E. (2005). Spurious chemical diffusion coefficients of Li^+^ in electrode materials evaluated with GITT. Electrochim. Acta.

[44] Chen W., Li G., Pei A., Li Y., Liao L., Wang H., Wan J., Liang Z., Chen G., Zhang H. (2018). A manganese-hydrogen battery with potential for grid-scale energy storage. Nat. Energy.

[45] Ilton E.S., Post J.E., Heaney P.J., Ling F.T., Kerisit S.N. (2016). XPS determination of Mn oxidation states in Mn (hydr)oxides. Appl. Surf. Sci..

[46] Gao X., Wu H., Li W., Tian Y., Zhang Y., Wu H., Yang L., Zou G., Hou H., Ji X. (2020). H^+^-insertion boosted α-MnO_2_ for an Aqueous Zn-ion battery. Small.

[47] Pan H., Shao Y., Yan P., Cheng Y., Han K.S., Nie Z., Wang C., Yang J., Li X., Bhattacharya P. (2016). Reversible aqueous zinc/manganese oxide energy storage from conversion reactions. Nat. Energy.

[48] Liu N., Wu X., Fan L., Gong S., Guo Z., Chen A., Zhao C., Mao Y., Zhang N., Sun K. (2020). Intercalation pseudocapacitive Zn^2+^ storage with hydrated vanadium dioxide toward ultrahigh rate performance. Adv. Mater..

[49] Zhang R., Liang P., Yang H., Min H., Niu M., Jin S., Jiang Y., Pan Z., Yan J., Shen X. (2022). Manipulating intercalation-extraction mechanisms in structurally modulated δ-MnO_2_ nanowires for high-performance aqueous zinc-ion batteries. Chem. Eng. J..

[50] Zuo Y., Liu P., Ling L., Tian M., Wang Z., Tian H., Meng T., Sun X., Cai S. (2022). Boosted H^+^ intercalation enables ultrahigh rate performance of the δ-MnO_2_ cathode for aqueous zinc batteries. ACS Appl. Mater. Inter..

[51] Pang Q., Sun C., Yu Y., Zhao K., Zhang Z., Voyles P.M., Chen G., Wei Y., Wang X. (2018). H_2_V_3_O_8_ nanowire/graphene electrodes for aqueous rechargeable zinc ion batteries with high rate capability and large capacity. Adv. Energy Mater..

[52] Wang X., Xi B., Ma X., Feng Z., Jia Y., Feng J., Qian Y., Xiong S. (2020). Boosting zinc-ion storage capability by effectively suppressing vanadium dissolution based on robust layered barium vanadate. Nano Lett..

[53] Cook J.B., Kim H.S., Lin T.C., Lai C.H., Dunn B., Tolbert S.H. (2017). Pseudocapacitive charge storage in thick composite MoS_2_ Nanocrystal-based electrodes. Adv. Energy Mater..

