# Supplementary material for: An Artificial MnWO4 Cathode Electrolyte Interphase Enabling Enhanced Electrochemical Performance of δ-MnO2 Cathode for Aqueous Zinc Ion Battery
Source: Materials (Basel). 2023 Apr 19;16(8):3228. doi: 10.3390/ma16083228 (PMC10141966; doi:10.3390/ma16083228)
Supplement: Supplementary file 1 [file materials-16-03228-s001.zip › materials-2331029-supplementary.pdf]

Supporting Information

An Artificial $MnWO_4$ Cathode Electrolyte Interphase Enabling Enhanced Electrochemical Performance of δ-MnO_2 Cathode for Aqueous Zinc Ion Battery

Hao Tian, Huanlin Zhang, You Zuo, Lei Ling, Tengfei Meng, Hang Zhang, Xiaohong Sun and Shu Cai *

Key Laboratory of Advanced Ceramics and Machining Technology of Ministry of Education, School of Materials Science and Engineering, Tianjin University, Tianjin 300072, China

* Correspondence: caishu@tju.edu.cn.

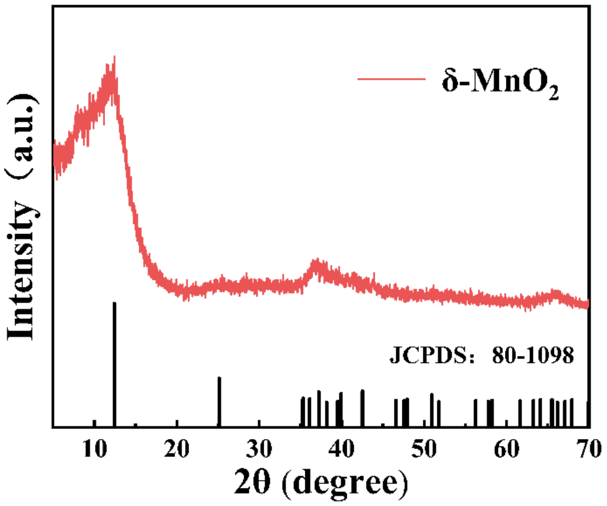

Figure S1 XRD pattern of synthetic δ-MnO_2 pristine powder.

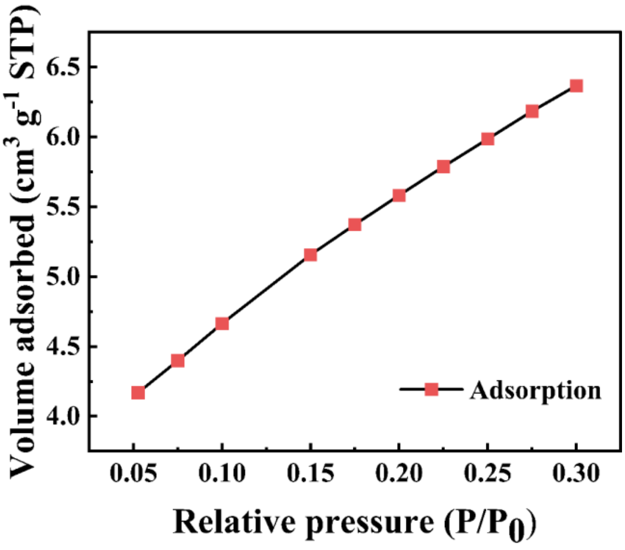

Figure S2 N_2 adsorption isotherm of δ-MnO_2

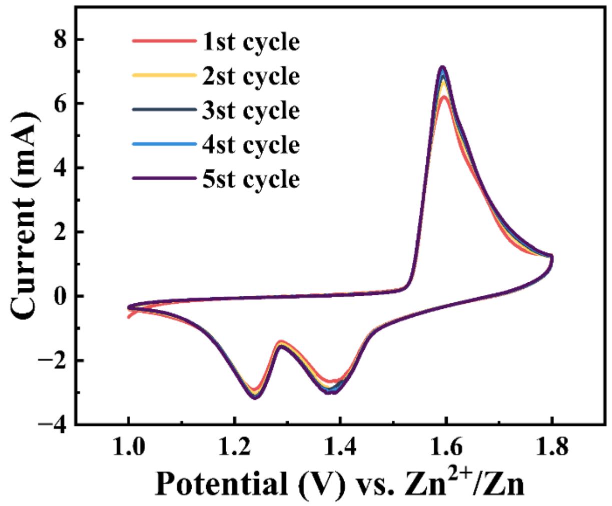

Figure S3 CV curves of the δ-MnO_2 electrode at 1 mV/s.

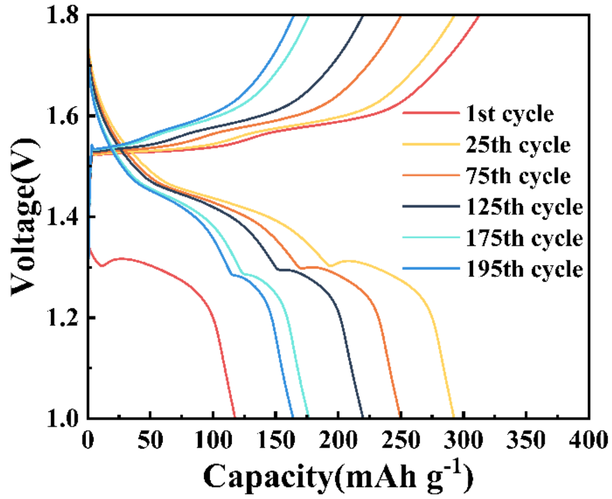

Figure S4 Galvanostatic charge and discharge curves of the δ-MnO_2 cathode at 0.2 A g^{-1}.

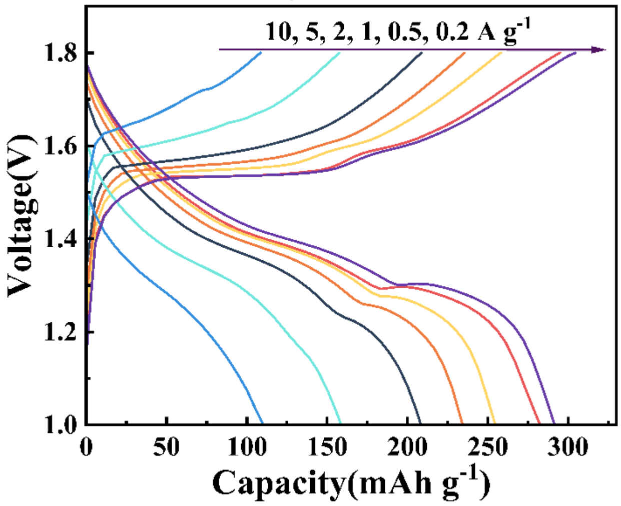

Figure S5 Galvanostatic charge and discharge curves of W-MnO_2 cathode at various current densities ranging from 0.2 A g^{-1} to 10 A g^{-1}.

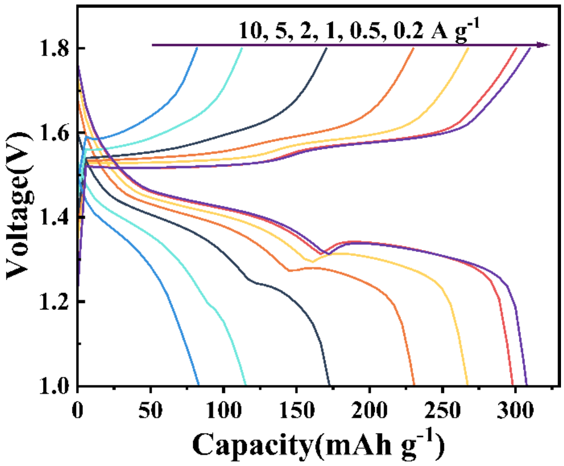

Figure S6 Galvanostatic charge and discharge curves of the δ-MnO_2 cathode at various current densities ranging from 0.2 A g^{-1} to 10 A g^{-1}.

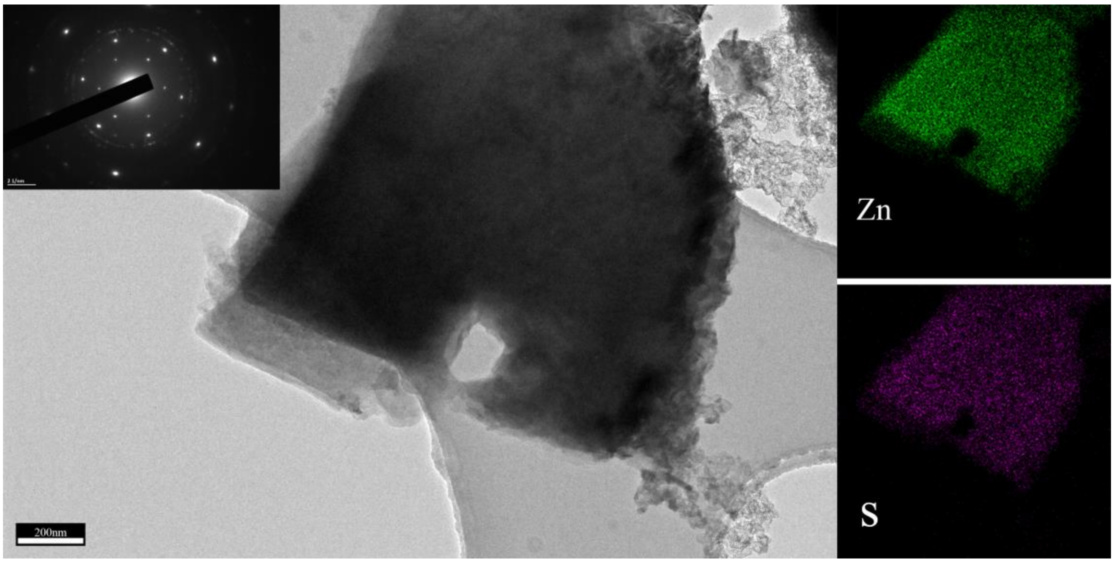

Figure S7 High-angle annular bright-field scanning TEM (HAABF-STEM) image

and the corresponding elemental mappings, respectively.

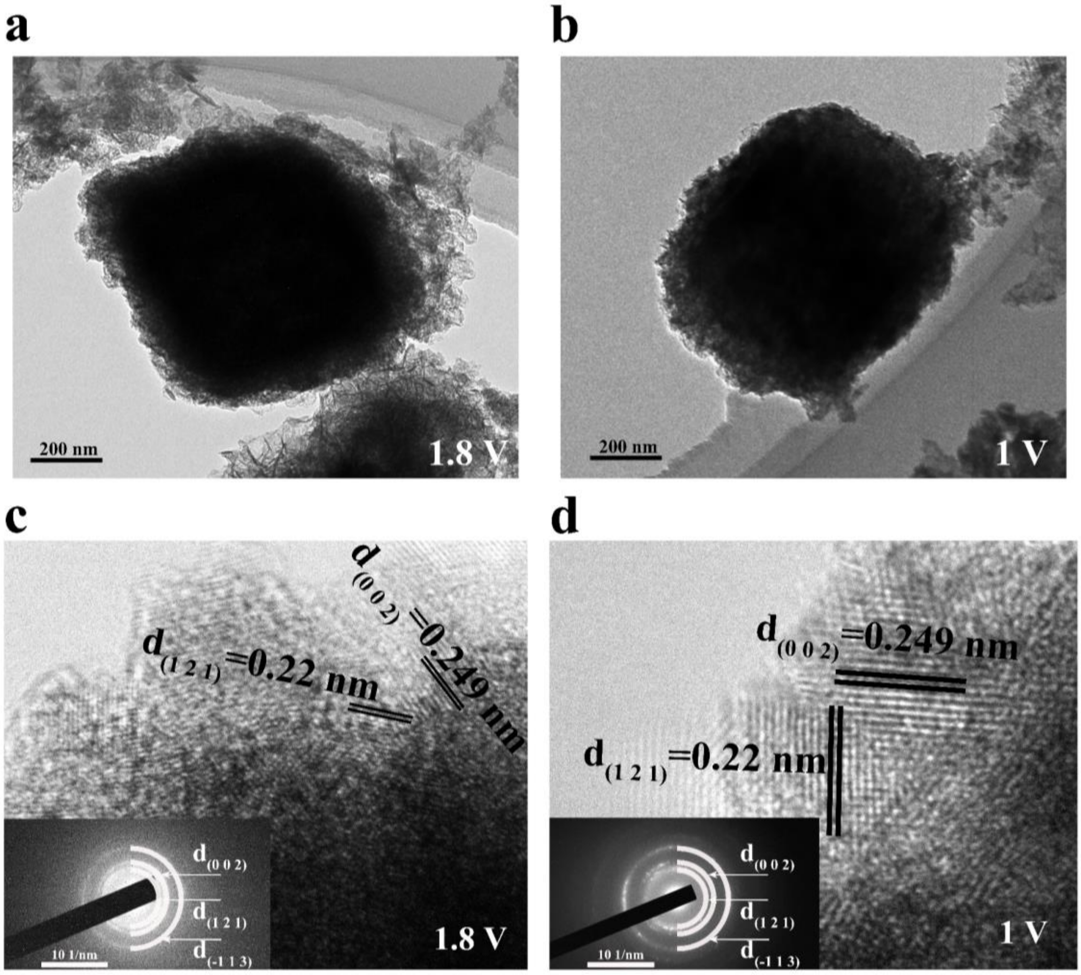

Figure S8 (a, b) TEM、HR-TEM and SAED pattern of W-MnO_2: (a,c) charged (1.8 V). (b,d) discharged (1 V).

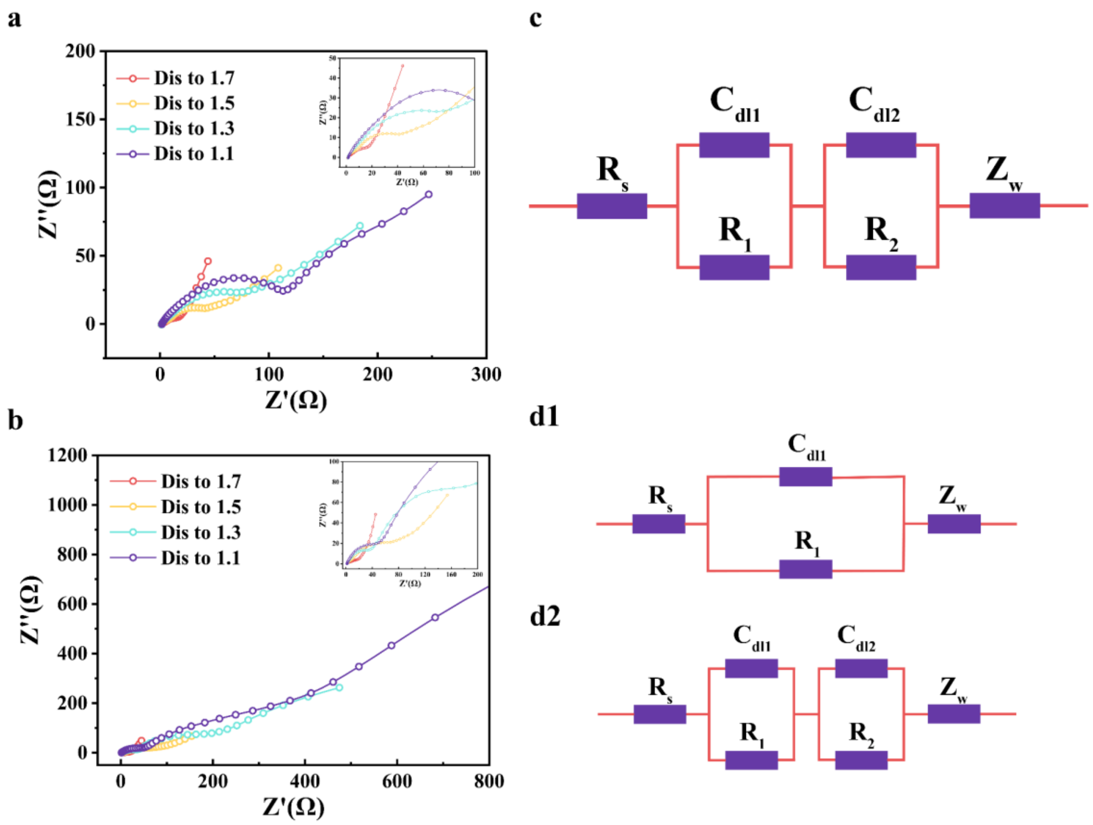

Figure S9 EIS of a) the W-MnO_2 and b) δ-MnO_2 cathodes at different stages during the discharge process. The equivalent circuit model for c) the W-MnO_2 and d) the δ-MnO_2 electrode.

The EIS data have fitted the data with the equivalent circuit, which is composed of two semicircles in the high and low-frequency regions, respectively. For δ-MnO_2 cathode, the EIS data show that the initial composition of one semicircle changes to two semicircles during the discharge process, which is attributed to the gradual production of basic zinc sulfate. The equivalent circuit of δ-MnO_2 at 1.7 V is shown in Figure S8d1. The equivalent circuit of δ-MnO_2 after 1.7 V is shown in Figure S8d2.

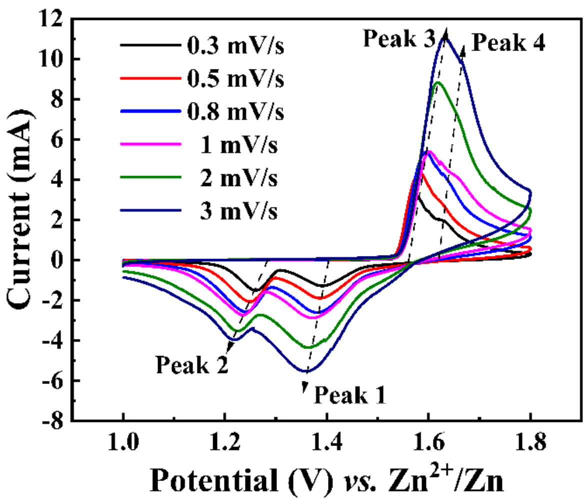

Figure S10 CV tests for δ-MnO_2 cathode at various scan rates ranging from 0.3 to 3 mV/s.

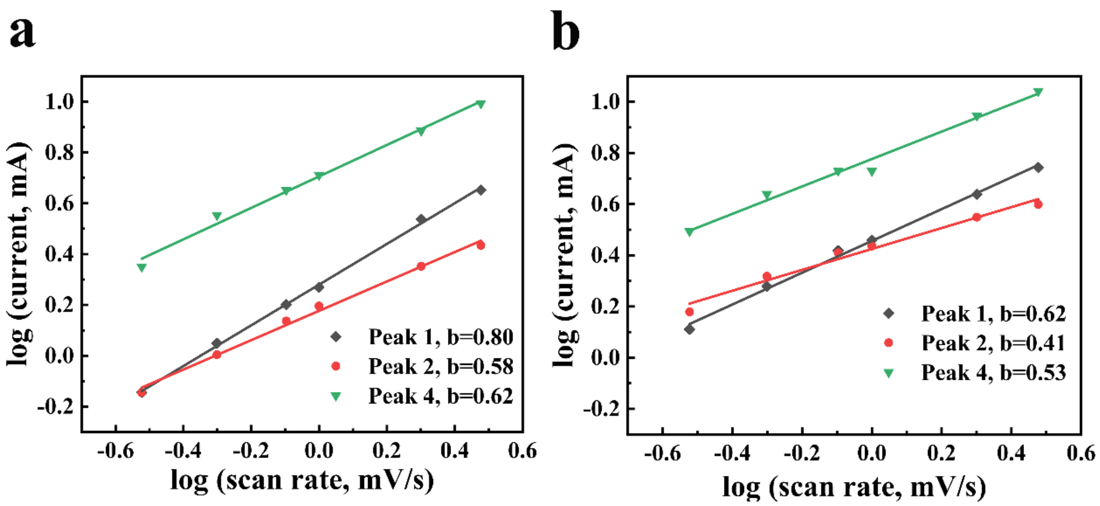

Figure S11 (a, b) b values of different peaks in CV curves for W-MnO_2 and δ-MnO_2 cathodes: (a) W-MnO_2. (b) δ-MnO_2.

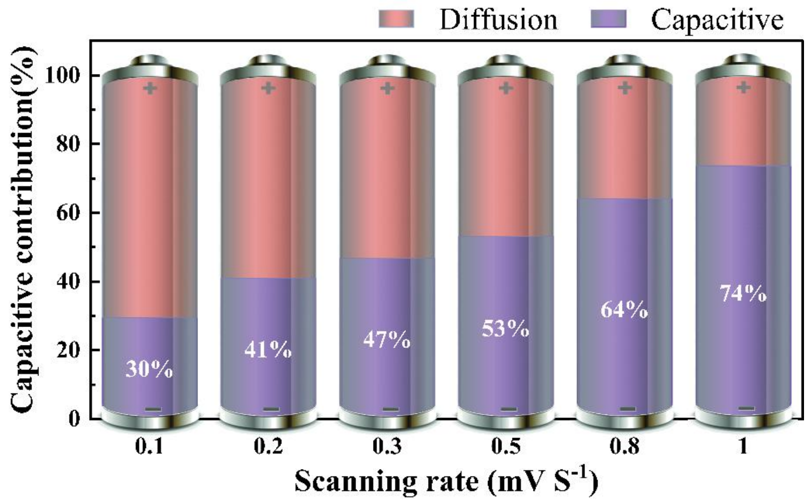

Figure S12 The proportion of capacitive and diffusion contributions at various scan

rates for δ-MnO_2.

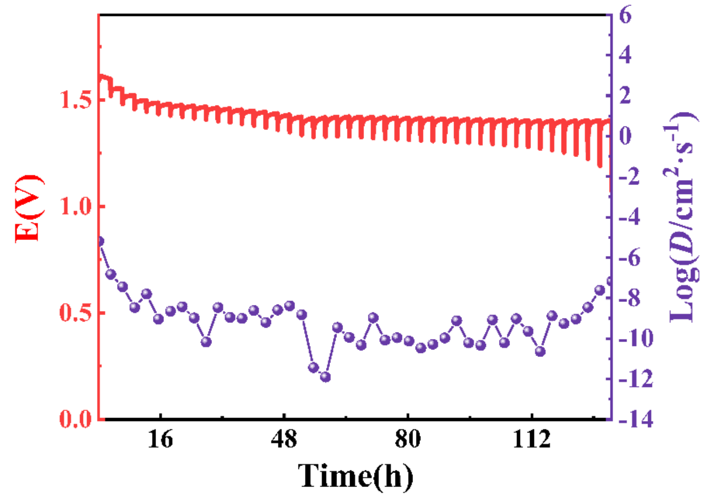

Figure S13 GITT test of the δ-MnO_2 cathode.

Table 1 Comparison of cycling performance with other recent studies

Electrode	Current density	Cycles	Capacity retention
Ca_2MnO_4 [1]	0.1 A g^{-1}	1000	80%
$Ba_{0.26}V_2O_5{\cdot}0.92H_2O$ [2]	5 A g^{-1}	2000	87%
MnO_2 [3]	3 A g^{-1}	3000	94%
$Zn_3V_2O_7(OH)_2{\cdot}2H_2O$ [4]	0.1 A g^{-1}	100	90%
MnO@C [5]	2 A g^{-1}	10000	70%
MnO_2 [6]	0.5 A g^{-1}	1000	82%
$Na_2V_6O_{16}{\cdot}3H_2O$ [7]	5 A g^{-1}	5000	85%
δ-MnO_2 (This work)	10 A g^{-1}	2000	98.2%

References

1. Guo, S.; Liang, S.; Zhang, B.; Fang, G.; Ma, D.; Zhou, J. Cathode interfacial layer formation via in situ electrochemically charging in aqueous zinc-ion battery. *Acs Nano* **2019**, 13, 13456-13464, doi:10.1021/acsnano.9b07042.
2. Luo, S.; Cao, X.; Su, Q.; Zhang, Y.; Liu, S.; Xie, X.; Liang, S.; Pan, A. Layered barium vanadate cathodes for aqueous zinc batteries: enhancing cycling stability through inhibition of vanadium dissolution. *Acs Appl Energ Mater* **2021**, 4, 6197-6204, doi:10.1021/acsaem.1c00979.
3. Wu, B.; Zhang, G.; Yan, M.; Xiong, T.; He, P.; He, L.; Xu, X.; Mai, L. Graphene scroll-coated α-MnO_2 nanowires as high-performance cathode materials for aqueous Zn-ion battery. *Small* **2018**, 14, 1703850, doi:10.1002/smll.201703850.
4. Guo, J.; Ming, J.; Lei, Y.; Zhang, W.; Xia, C.; Cui, Y.; Alshareef, H.N. Artificial solid electrolyte interphase for suppressing surface reactions and cathode dissolution in aqueous zinc ion batteries. *Acs Energy Lett* **2019**, 4, 2776-2781, doi:10.1021/acsenergylett.9b02029.

5. Li, S.; Yu, D.; Liu, L.; Yao, S.; Wang, X.; Jin, X.; Zhang, D.; Du, F. In-situ electrochemical induced artificial solid electrolyte interphase for MnO@C nanocomposite enabling long-lived aqueous zinc-ion batteries. *Chem Eng J* **2022**, 430, 132673, doi:10.1016/j.cej.2021.132673.
6. Liu, Y.; Zhi, J.; Hoang, T.K.A.; Zhou, M.; Han, M.; Wu, Y.; Shi, Q.; Xing, R.; Chen, P. Paraffin Based cathode-electrolyte interface for highly reversible aqueous zinc-ion battery. *Acs Appl Energ Mater* **2022**, 5, 4840-4849, doi:10.1021/acsaem.2c00252.
7. Zhang, L.; Zhang, B.; Hu, J.; Liu, J.; Miao, L.; Jiang, J. An in situ artificial cathode electrolyte interphase strategy for suppressing cathode dissolution in aqueous zinc ion batteries. *Small Methods* **2021**, 5, 2100094, doi:10.1002/smtd.202100094.
